# iTBS over the left hV6A enhances PPC-PPC functional connectivity during reaching tasks: an EEG study

**DOI:** 10.3389/fnins.2025.1536308

**Published:** 2025-03-26

**Authors:** Jing Chen, Qian Ding, Ya-wen Li, Yu-hong Huang, Shan-tong Yao, Ri-yu Guo, Long-ping Wang, Xin-hua Wei, Yue Lan, Guang-qing Xu

**Affiliations:** ^1^School of Rehabilitation Medicine, Shandong Second Medical University, Weifang, China; ^2^Department of Rehabilitation Medicine, Guangdong Provincial People's Hospital (Guangdong Academy of Medical Sciences), Southern Medical University, Guangzhou, China; ^3^Department of Rehabilitation Medicine, Guangdong Cardiovascular Institute, Guangdong Academy of Medical Sciences, Guangzhou, China; ^4^Department of Radiology, Guangzhou First People's Hospital, School of Medicine, South China University of Technology, Guangzhou, China; ^5^Department of Rehabilitation Medicine, Guangzhou First People's Hospital, School of Medicine, South China University of Technology, Guangzhou, China

**Keywords:** transcranial magnetic stimulation, posterior parietal cortex, functional neuroimaging, reaching, V6A

## Abstract

**Introduction:**

The functional connectivity of the posterior parietal cortex-primary motor cortex (PPC-M1) is involved in goal-directed reaching actions and integrating visuomotor transformation. Human area V6A (hV6A), located in the medial PPC, is a critical node of the dorsomedial system that is involved in targeting during reaching movements. Here, we used Electroencephalography (EEG) to investigate functional connectivity and network efficiency during right-hand reaching tasks after inducing left hV6A activity with intermittent theta burst stimulation (iTBS).

**Methods:**

Based on individualized MRI neural navigation, 23 healthy subjects were randomly accepted into either real left hV6A or sham iTBS on 2 days. Resting-state and goal-directed reaching task EEG were recorded at baseline and immediately after iTBS to assess the effects of iTBS on functional connectivity. Alongside the reaching task, an additional Stroop test was conducted to assess each participant’s degree of attention.

**Results:**

In the alpha band, medial posterior parietal cortical interhemispheric functional connectivity significantly increased during right-hand reaching tasks after hV6A iTBS (*p =* 0.008) but not after sham iTBS (*p =* 0.726). Alpha and beta bands small-worldness of right-hand reaching tasks significantly increased (*p =* 0.001 and 0.013, respectively) but not after sham iTBS (*p =* 0.915 and 0.511, respectively).

**Discussions:**

Functional connectivity of the bilateral PPC and functional network efficiency increased after iTBS of the left hV6A during right-hand reaching tasks. These findings indicate that the left hV6A should be a potential target for iTBS modulation to improve the orienting movement function in space.

## 1 Introduction

A goal-directed reaching action can be described as the transport of the hand by the upper limb to an object. Located on the medial parieto-occipital cortex of the macaque monkey, the bimodal visual and somatosensory area V6A is integral to the reaching action (Galletti et al., 2003; Fattori et al., 2015; Goldenkoff et al., 2021). Homologous with the cortex of the macaque monkey V6A, in the human brain, the human area V6A (hV6A) (Pitzalis et al., 2013; Pitzalis et al., 2015) is a critical cortical node of the dorsomedial system (Vesia et al., 2010), which is a major parietal network system involved in the motor control of the upper limb. hV6A is equally closely related to two processes of the reaching action, including programming [motor intention, including the initial movement parameters of the reach (Verhagen et al., 2008)] and the online control of movements (Striemer et al., 2011; Breveglieri et al., 2023a). Moreover, the left parietal hemisphere is more dominant in movement intention (Rushworth, 2003) and motor control (Goodale, 1988). Thus, the left hV6A has been extensively investigated in recent reaching experiments (Breveglieri et al., 2023a; Breveglieri et al., 2021a; Breveglieri et al., 2021b; Breveglieri et al., 2023c; Breveglieri et al., 2023b).

The connectivity of PPC-M1 and PPC-PPC influences the upper limb motor activity in healthy elders and stroke survivors (Goldenkoff et al., 2021; Hensel et al., 2023). Interestingly, recent studies demonstrated that repetitive transcranial magnetic stimulation (rTMS) of specific subregions of the PPC, omitting hV6a, can change the cortico-cortical inter- and intra-hemispheric connectivity (Mazzi et al., 2024; Nyffeler, 2019). Furthermore, Nyffeler (Nyffeler, 2019) studies showed that rTMS not only modulates parietal cortical excitability but also promotes the functional recovery of survivors with cerebral lesions. Meanwhile, recordings in other parietal cortices after rTMS indicated subsequent functional network changes concomitant to behavioral performance changes (Schintu et al., 2021). Whether rTMS of the left hV6A would produce similar effects on the functional connectivity of PPC-M1 and PPC-PPC, and even change functional networks to improve reaching ability, has never been investigated.

In previous studies, the connection between hV6A and M1 using dual-site paired-pulse transcranial magnetic stimulation was functionally specific to arm transport but is still under debate [inhibitory (Breveglieri et al., 2023b) and facilitatory (Vesia et al., 2013)]. This might be due to the different tasks and neuronavigational methods, which include MRI-based TMS over the superior parieto-occipital cortex SPOC area [also including area hV6A (Vesia et al., 2013)] or TMS over the Talairach coordinates of the target hV6A (Breveglieri et al., 2023b). MRI-based rTMS over specific subregions of the PPC increases the behavioral effect size (Sack et al., 2009) and takes into account the interindividual variance in neuroanatomy within the superior parietal cortex (Scheperjans et al., 2007).

Intermittent theta burst stimulation (iTBS), a specific pattern of rTMS, has been used as a neural modulation approach with greater spatial accuracy and efficacy than other tools (such as HD-tDCS) over subregions of the PPC (Gan et al., 2019). Meanwhile, Goldenkoff et al. (2021) reported that poor upper limb motor performance in the elderly is associated with a reduced role of the PPC in driving the M1. As the “upstream” of the M1 (Mathew et al., 2017; Goldenkoff et al., 2023), improving the excitability of the hV6A appears to be a feasible method to enhance limb performance. iTBS impacts functional networks in brain areas remote from the stimulated site. It was confirmed indirectly with monkey and human studies that the connection between hV6a and M1 is anatomical (Tosoni et al., 2014; Gamberini et al., 2009; Passarelli et al., 2011). Electroencephalography (EEG) is a tool used widely to measure neurophysiological changes and has provided useful information in previous parietal studies (Karabanov et al., 2012; Verhagen et al., 2013; Rocha et al., 2018; Rizk et al., 2013). Current evidence indicates that the preparation and execution of upper limb movements are associated with changes in alpha and beta bands that can be recorded by EEG (Storti et al., 2016; Tzagarakis et al., 2010; Hsu et al., 2016). Phase changes of frequency bands refer to synchrony of cortical activity in anatomically distinct but functionally collaborating brain regions, which forms the basis of a functional brain network (Ismail and Karwowski, 2020). Small-worldness is a graph theory analysis that reflects the functional network’s overall balance and efficiency through the ratio of cortical clustering and path length (Bullmore and Sporns, 2009; Caliandro et al., 2021).

In the present study, we used resting-state and reaching-state EEG to investigate the effects of MRI-based iTBS over left hV6A. The right parietal hemisphere is the critical area for visuospatial attention (Rushworth, 2003) and corpus callosum connection between PPCs (Schintu et al., 2021). To investigate possible indirect TMS effects of right PPC, we novelly added the left-hand reaching task (right hV6A-M1) and the Stroop color and word test. We anticipated that (Galletti et al., 2003) the functional connectivity of the left PPC-M1 and PPC-PPC would increase during the resting state and right-hand reaching state after iTBS of the left hV6A, and (Fattori et al., 2015) there would be an improvement in right-hand reaching performance, as well as an increase in network efficiency.

## 2 Methods

### 2.1 Participants

Twenty-six healthy right-hand subjects with normal or corrected vision participated in the study. Following screening for medical contra-indications to MRI and TMS, three subjects were excluded due to poor raw data, leaving 23 participants for analysis. They included 12 males and 11 females with an age range of 21–25 years. The sample size was based on Dr. Breveglieri’s hV6A studies in the last 5 years (Breveglieri et al., 2023a; Breveglieri et al., 2021a; Breveglieri et al., 2021b; Breveglieri et al., 2023c; Breveglieri et al., 2023b; Breveglieri et al., 2025). Subjects gave their written informed consent for the experimental procedures that were approved by the Guangdong Provincial People’s Hospital Human Research Ethics Committee (KY2024-234-01). The study was performed in accordance with the Declaration of Helsinki.

### 2.2 Experimental design

**Figure 1:**
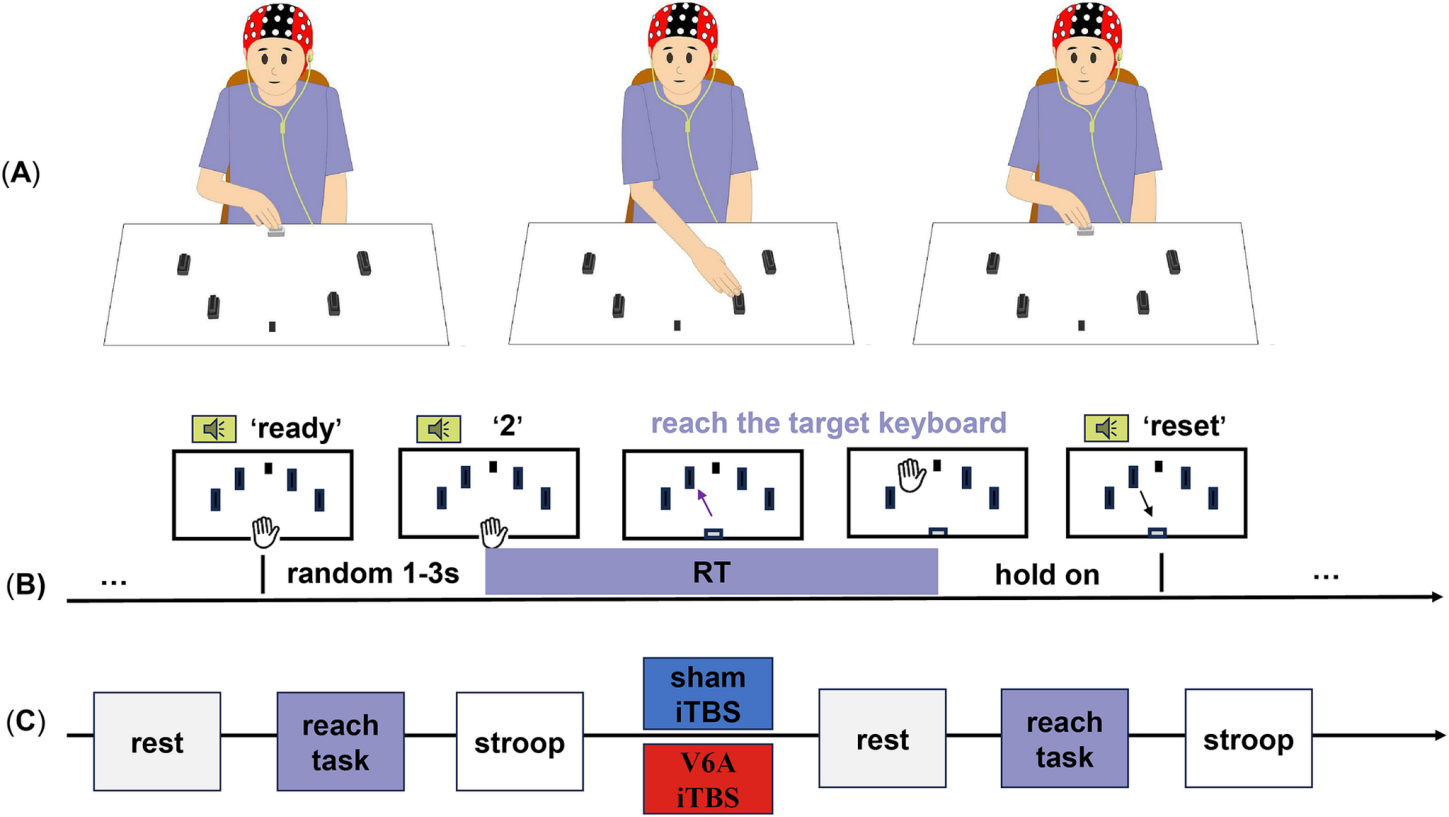
Experimental design and procedure of the goal-directed reaching tasks. The participants were randomly assigned to two iTBS protocols (blue for sham iTBS, red for real iTBS of the left hV6A) on different days. **(A)** Four target keyboards (black) were placed on a table around a center fixation (a black sticker) and a HOME keyboard (white). **(B)** After hearing the ‘direction’ from the voice prompt, the projectile’s range (straight line without stopping in the middle) to the corresponding target keyboard was arranged. The black keyboards from left to right represent ‘1’,’2’,’3’,and ‘4’. The keyboard was pressed and held until the ‘reset’ prompt indicated a return to the HOME keyboard, which ended this trial. **(C)** The resting-state EEG, reaching-task EEG, and Stroop test were successively recorded before and after modulation.

All subjects participated in two stimulation protocols, each randomized to receive experimental (real iTBS on left hV6A) and control (sham iTBS) stimulation on two separate days. They were separated by at least 10 days. EEG activities were immediately recorded before and after the stimulation protocols (Figure 1C).

### 2.3 Individualized neuronavigation and intermittent theta burst stimulation

**Figure 2:**
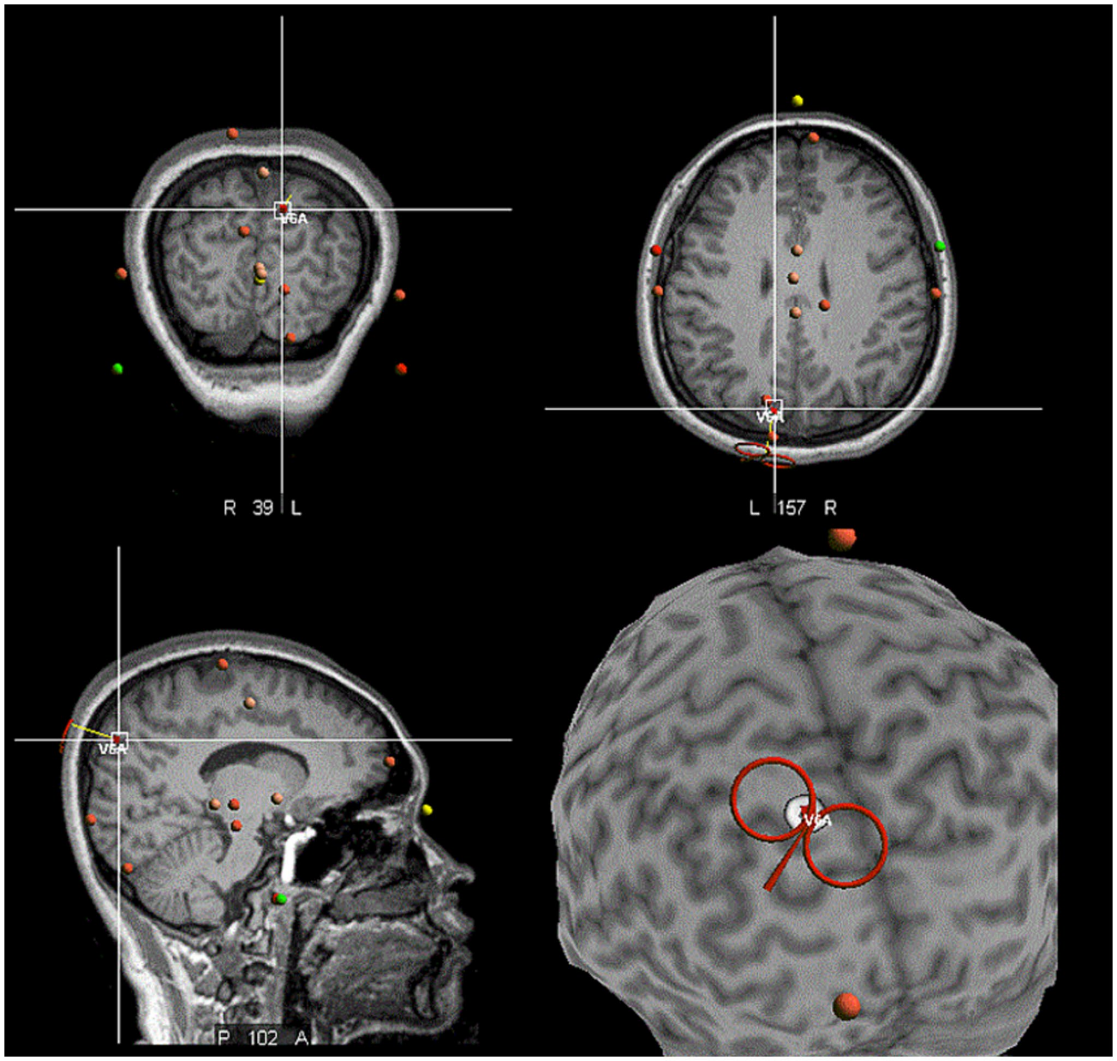
The location of the hV6A in the neuronavigational system. The red coil against the target left hV6A, which was located in the medial parieto-occipital cortex.

The site of the hV6A was identified using structural MRI data of each participant. In the neuronavigation system (Visor2, ANT Neuro, Hengelo, the Netherlands), the following positioning was performed in sequence: Nasion marker, left and right ear marker, followed by the three-point positioning (anterior and posterior commissure, inter-hemispheric point), the AC-PC line positioning, and the Talairach coordinate system markers. The scalp, skull, and brain were divided to create individualized three-dimensional head models. The Talairach coordinates of the target hV6A (*x* = −10, *y* = −78, *z* = 40) and its network were set (Figure 2). These coordinates were the same as those used in previous TMS studies on hV6A (Breveglieri et al., 2023a; Breveglieri et al., 2021a; Breveglieri et al., 2021b; Breveglieri et al., 2023c; Breveglieri et al., 2023b) and similar to those used in the SPOC study (Vesia et al., 2010; Vesia et al., 2013). iTBS was applied using an NS5000 magnetic stimulator (YIRUIDE Medical Co, Wuhan, China). Surface electromyography (EMG) was recorded from the first dorsal interosseous (FDI) in the right hand. The iTBS pattern consisted of bursts containing three pulses at 50 Hz and repeated at 5 Hz. A 2 s train of TBS was repeated every 10 s for a total of 192 s (600 pulses in total) (Huang et al., 2005). The iTBS protocol was 80% active motor threshold (AMT). A neuronavigational system was used to ensure reliable and consistent coil positioning over the hotspot throughout the experiment. Coil position error was controlled at <5 mm displacement and ≤ 3° relative to the target (Ding et al., 2021; Ding and Patten, 2018). In the sham stimulation, the coil was placed perpendicular to the scalp, ensuring no magnetic field passed through the scalp.

### 2.4 EEG

#### 2.4.1 EEG acquisition

EEG signals were recorded using a TMS-compatible EEG cap (ANT Neuro, Enschede, Netherlands) with 64 Ag/AgCl electrodes. All channels were referenced online to CPz and amplified with an eego amplifier (ANT Neuro, Enschede, the Netherlands). Data were sampled at 2,048 Hz with impedance kept below 10 kΩ for all channels throughout data collection.

##### 2.4.1.1 Resting state

Three minutes of resting EEG activity was recorded immediately before and after the stimulation protocol. Participants sat in a comfortable chair with their eyes closed.

##### 2.4.1.2 Goal-directed reaching tasks

Subjects sat comfortably at a distance of 20 cm from the table. With their shoulders in a neutral position and their forearm internally rotated, the test hand (including the middle, index, and ring fingers) was held down and placed on the HOME keyboard. Their other hand was placed flat on their knee. The four black keyboards (length: 4.5 cm; width: 2.8 cm; height: 2.5 cm; effective pressure area: 3.9 cm^2^) were placed at 30°, 60°, 120°, and 150° around the center of the HOME keyboard. The fixation point was at 90°. Previous studies have shown that the hV6A is activated only when the peripheral target arrives. Therefore, our experimental design did not include the central target (Breveglieri et al., 2021a). The auditory input provided new spatial information and prompted subjects to roughly stretch in the direction and move according to voice guidance. Subjects were asked to gaze at the fixation point, listen to voice prompts, and complete tasks set by the E-prime, reducing head movement and blinking. The ‘direction’ prompted subjects to leave the HOME keyboard immediately, reach out, and pat the corresponding target keyboard. The ‘direction’ (Galletti et al., 2003; Fattori et al., 2015; Goldenkoff et al., 2021; Pitzalis et al., 2013) represented four directions from left to right. The ‘reset’ prompted the subjects to return their hand to the initial position. These two prompts were separated by 4 s. The reset phase lasted for 5 s to ensure that the subject had enough time to reset and be in a stable state for the next trial. There was a ‘ready’ prompt at the end of the reset phase. Then, the next ‘direction’ was given after a randomization interval of 1–3 s. Each direction was presented 12 times, resulting in a total of 48 random trials for each hand side, with a rest period every 24 trials to avoid muscle fatigue (Figures 1A,B). Auditory information reached the PPC in <40 ms. A reaction time (RT) above 40 ms means the reaching action, allowing for the prompt (Koch et al., 2008).

#### 2.4.2 EEG data analysis

Acquired EEG signals were analyzed off-line using MATLAB2019b. EEGLAB toolbox (version 14.1.2b) was used for EEG data preprocessing. The signals of raw data were sampled down to 1,024 Hz. The resting-state EEG was filtered by a band-pass filter with cutoffs ranging from 0.1 to 40 Hz and segmented into epochs ranging from 0 to 180 s. The reaching-task EEG was filtered with cutoffs ranging from 1 to 30 Hz, segmented into epochs ranging from −1 to 2 s, and calibrated to a baseline from −1 to 0 s. The independent component analysis (ICA) was performed to exclude components endowing eye (blinking and movement), cardiac, and muscle artifacts. The resulting data were inspected to exclude remaining “bad trials” (i.e., amplitudes >100 mV) and re-referenced using the average signals from each scalp electrode as a reference.

We used the phase locking value (PLV) to measure functional connectivity (Lachaux et al., 1999). The PLV is a measure of synchronization in the time domain, and the definition of the single-trial formula is as follows:


$$PLV\left(t\right)=\frac{1}{N}\left|\overset{N}{\underset{n=1}{\sum }}\exp\left(j\Delta \varphi \left(t\right)\right)\right|$$

where t is the specific time point, N is the number of sample points, and $\Delta \varphi \left(t\right)$ is the difference of instantaneous phases between electrode pairs at time t (Xu et al., 2021; Benzy et al., 2020). According to the MATLAB script, the PLV of the electrodes included in the brain region of interest was calculated in alpha and beta bands. The calculated PLV was converted by fisher-z, and the functional connectivity among the brain regions was obtained by averaging the four brain regions of interest [Left M1: FC1, FC3, FC5, C1, C3, C5; Right M1: FC2, FC4, FC6, C2, C4, C6; Left PPC: P1, P3, PO3; Right PPC: P2, P4, PO4 (Whybird et al., 2021; Ciavarro et al., 2013)].

GRaph thEoretical Network Analysis (GRETNA) toolbox was used for graph theory analysis. In the present study, weighted and undirected networks were built based on the PLV of goal-directed reaching tasks described above (Xu et al., 2021). We constructed functional brain networks over the whole range of costs (0.10–0.40) at an interval of 0.05 using a weighted matrix (Ding et al., 2021). The formula of small-worldness is as follows:


$$Sw=\frac{Cw/Cw_{\mathrm{rand}}}{Lw/Lw_{\mathrm{rand}\text{.}}}$$

where Cw is the mean weighted clustering coefficient, and Lw is the mean weighted characteristic path length. ‘Small worldness’ (sigama) is expressed as a quotient of Cw and Lw , normalized to the values of $Cw_{\mathrm{rand}}$ and $Lw_{\mathrm{rand}}$ found in an equivalent weighted random network (Caliandro et al., 2021; Onoda and Yamaguchi, 2013).

### 2.5 The Stroop color and word test and Stroop effect analysis

The Stroop test is a neuropsychological test used for experimental and clinical purposes. It has also been used to assess changes in attention induced by rTMS (Parris et al., 2021). Subjects sat in front of a screen programmed by the E-Prime experimental software and pressed the corresponding keyboard according to the colors of the word presented. Red, yellow, blue, and green corresponded to F, G, H, and J keys (red ‘green’ press F key, red ‘red’ press F key). A total of four colors (red, yellow, blue, green), four color-words (‘red’, ‘yellow’, ‘blue’, ‘green’), word color consistency, and word color inconsistency accounted for half of the total. Subjects’ reaction time and accuracy were recorded. There was a total of 144 trials with three breaks to avoid fatigue. We calculated the trials that responded correctly, and the Stroop effect equation is as follows:


$$\mathrm{Stroop effect}=RT_{\mathrm{inconsistent}}-RT_{\mathrm{consistent}}$$

### 2.6 Statistical analysis

Repeated measures ANOVA was conducted to the absolute and relative error with the within-subject factors. The criteria for evaluating the spherical hypothesis by Mauchly’s test and the Greenhouse–Geisser procedure were modified. The data normality and homoscedasticity were previously guaranteed by the Levene and Shapiro–Wilk tests.

For behavioral indicators and small-worldness, we performed a two-way repeated measures ANOVA model [Stimulation (2) × Timepoint (2)]. For reaching tasks, we performed a three-way repeated measures ANOVA model [Stimulation (2) × Timepoint (2) × ROI (3)]. The ROIs of left-hand tasks included LPPC-RM1, RPPC-RM1, and LPPC-RPPC. The ROIs of the right-hand tasks included LPPC-LM1, RPPC-LM1, and LPPC-RPPC. Significant three-way interactions were investigated by one-way repeated measure ANOVAs followed by *post-hoc* tests with Bonferroni correction (Rocha et al., 2018).

## 3 Results

### 3.1 Behavioral indicators

The repeated measures ANOVA model revealed that there were no significant interactions of reaching RTs [left hand: *F*_(1,22)_ = 0.83, *p =* 0.370; right hand: *F*_(1,22)_ = 1.81, *p =* 0.192], nor was there a significant Stroop effect [*F*_(1,22)_ = 0.22, *p =* 0.641] (Table 1).

**Table 1:** The reaching task and Stroop test results.

		Sham		hV6A	
		Baseline	after iTBS	Baseline	after iTBS
Reaching task RT	L	1190.2609±179.86238	1207.1739±167.99909	1193.6087±198.88456	1228.3478±200.56616
	R	1177.6957±156.85907	1182.6087±161.46284	1179.5217±188.85321	1213.6522±197.16412
Stroop effect		118.7391±63.24715	119.9565±63.89443	123.6957±64.97442	133.0870±62.92847

The first two rows: there were not significantly increase in RTs of the left and right-hand tasks, respectively (Mean ± standard deviation, unit: ms). The last row: Stroop effect have not significant difference. RT indicates reaction time of reaching task, L indicates left-hand reaching, R indicates right-hand reaching.

### 3.2 Resting-state functional connectivity

**Figure 3:**
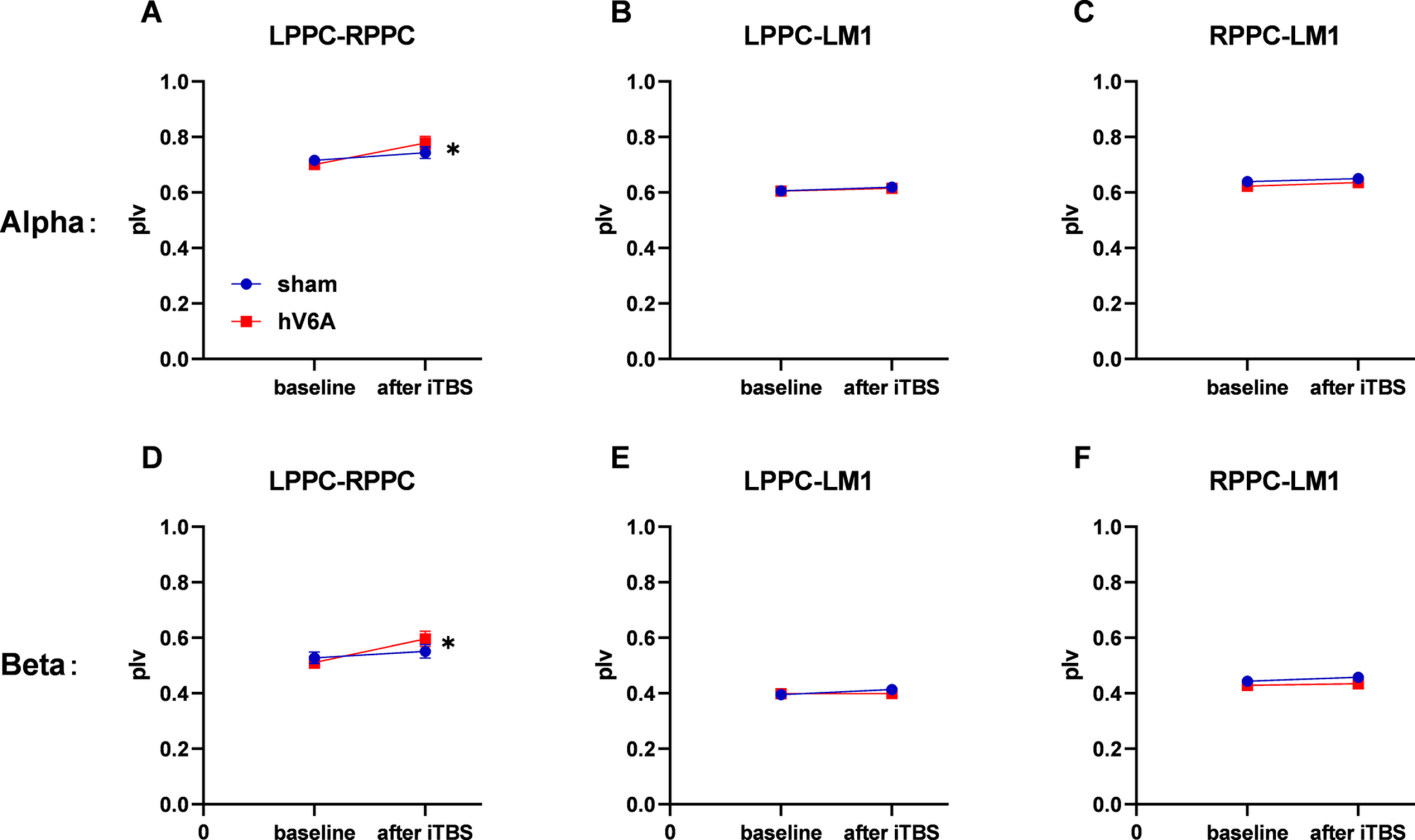
Alpha and beta bands functional connectivity measured at rest. **(A–C)** The alpha band LPPC-LM1, RPPC-LM1, and LPPC-RPPC functional connectivity, respectively. **(D–F)** The three types of beta band functional connectivity. The alpha and beta bands functional connectivity of LPPC-RPPC significantly increased after iTBS without stimulation protocol difference. * indicates a significant difference (*p* < 0.05). The blue line indicates sham iTBS and the red line indicates real iTBS over the left hV6A.

In the alpha and beta bands, the repeated measures ANOVA model revealed significant Timepoint × ROI interaction [alpha: *F*_(1,22)_ = 3.33, *p* = 0.045; beta: *F*_(1,22)_ = 5.18, *p* = 0.009], while there was no significant main effect of stimulation type. *Post hoc* revealed that LPPC-RPPC functional connectivity in both alpha and beta bands was increased after either real or sham iTBS (*p* = 0.018 and 0.003, respectively). There were no significant changes in other pairs of functional connectivity (*p* > 0.05) (Figure 3).

### 3.3 Reaching task functional connectivity

#### 3.3.1 Left hand

The repeated measures ANOVA model revealed no significant interaction.

#### 3.3.2 Right hand

**Figure 4:**
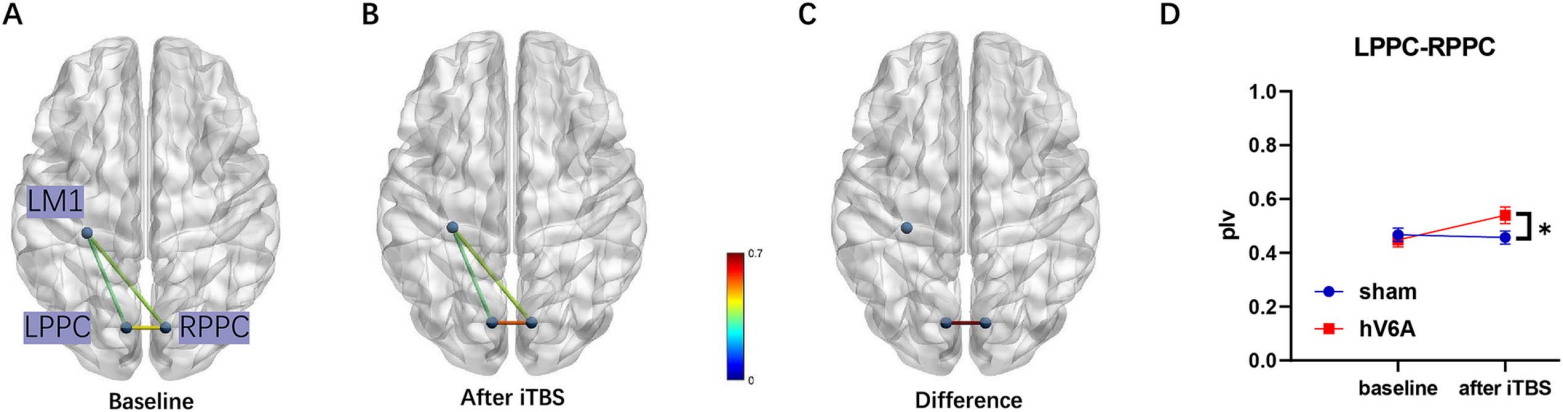
Alpha band functional connectivity measured during right-hand reaching tasks. **(A)** The functional connectivity situation of LPPC-LM1, RPPC-LM1, and LPPC-RPPC before real iTBS. **(B)** The same functional connectivity situation after real iTBS. The functional connectivity of LPPC-RPPC **(C)** increased after real iTBS over the left hV6A compared to the sham modulation **(D)**. * indicates a significant difference (*p* < 0.05).

In the alpha band, the three-way repeated measures ANOVA model revealed a significant interaction. The Stimulation × Timepoint interaction [*F*_(1,22)_ = 6.48, *p* = 0.018] of the LPPC-RPPC functional connectivity was significant, and *post hoc* analysis revealed that the functional connectivity of PPCs was increased after real iTBS over the left hV6A (*p* = 0.008) compared to the sham iTBS (*p* = 0.726). The ROI × Timepoint interaction of real iTBS over the left hV6A was significant [*F*_(1,22)_ = 4.96, *p* = 0.024], and *post hoc* analysis revealed that the functional connectivity of PPCs was increased after iTBS compared to the baseline (*p* = 0.008). This did not include other pairs of functional connectivity (*p* > 0.05). The ROI × Stimulation interaction after iTBS was significant [*F*_(1,22)_ = 5.49, *p* = 0.012], and *post hoc* analysis revealed that the functional connectivity of PPCs was increased with real iTBS over the left hV6A (*p* = 0.006) compared to the sham iTBS, not including other pairs of functional connectivity (*p* > 0.05) (Figure 4). *p*-value was adjusted using the Bonferroni correction.

In the beta band, the Stimulation × ROI main effect was significant [*F*_(1,22)_ = 3.663, *p* = 0.034]. However, no significant changes were revealed *post hoc*.

### 3.4 Small-worldness

**Figure 5:**
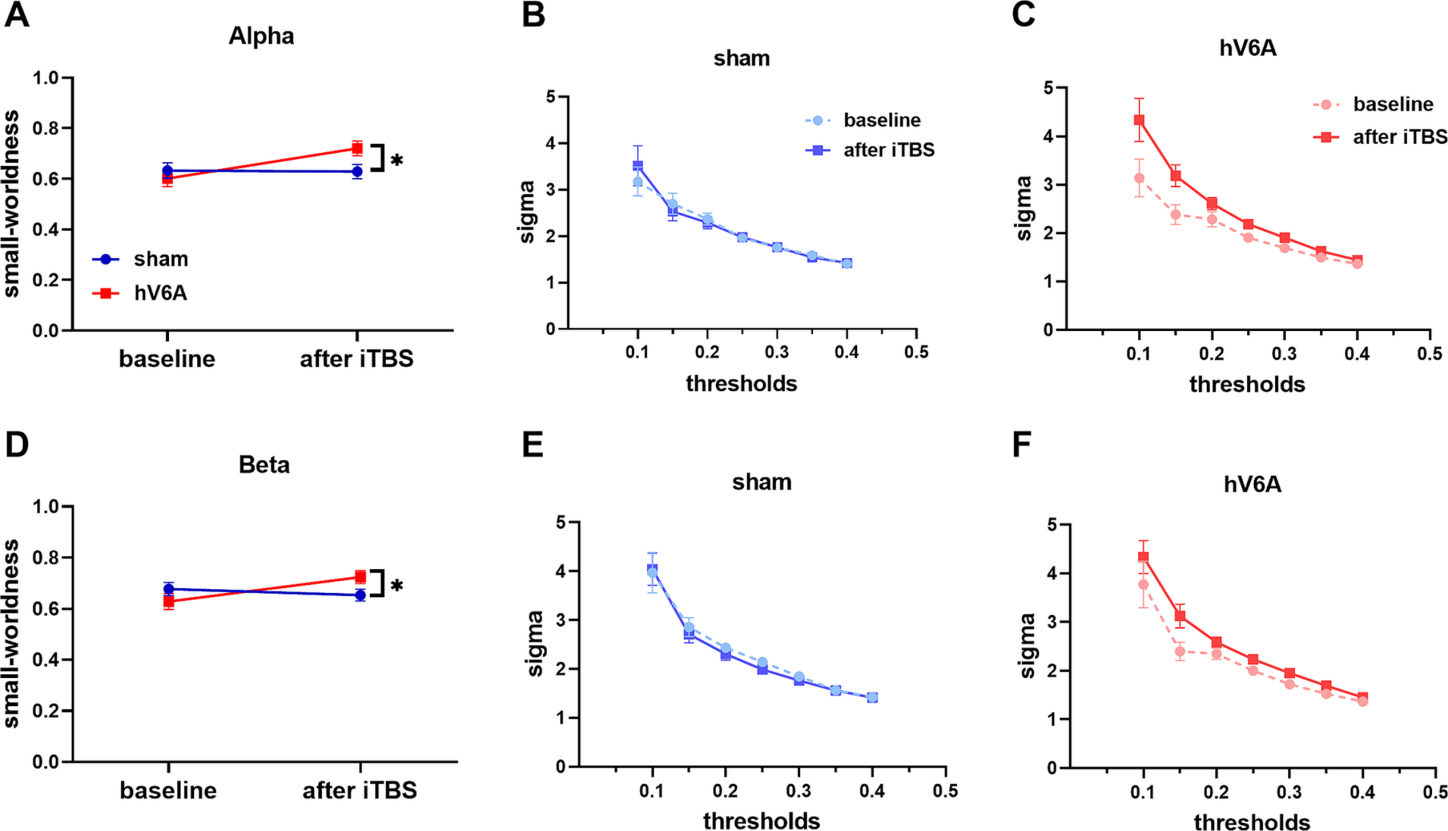
Small-worldness of right-hand reaching tasks. ‘sigma’ stands for small-worldness of alpha **(A)** and beta **(D)** bands. The network efficiency of these bands increased after real iTBS over the left hV6A compared to the sham. The area under the curve (AUC) represents the situation of alpha **(B,C)** and beta **(E,F)** bands sigma at different sparsity thresholds in the range 0.1–0.4. * indicates a significant difference (*p* < 0.05).

The repeated measures ANOVA model revealed a significant interaction of alpha and beta bands [alpha: *F*_(1,22)_ = 5.71, *p =* 0.026; beta: *F*_(1,22)_ = 4.68, *p =* 0.042]. Small-worldness was significantly increased after real iTBS over the left hV6A: *p =* 0.001; beta: *p =* 0.013) but not with sham iTBS (alpha: *p =* 0.915; beta: *p =* 0.511) (Figure 5).

## 4 Discussion

In this study, we demonstrated the results of a clinical study on functional connectivity and network efficiency changes after iTBS over the left hV6A in healthy individuals. The main findings of the present study are as follows: (1) Both the alpha and beta bands of the LPPC-RPPC functional connectivity at rest increased significantly after either sham or real iTBS; (2) Alpha band functional connectivity of bilateral PPCs during right-hand reaching was increased significantly after real iTBS; (3) Both alpha and beta bands functional network efficiency during right-hand reaching was increased significantly after real iTBS. In summary, the results were consistent with our expectations. iTBS over the left hV6A could enhance functional connectivity and network efficiency during right-hand reaching.

### 4.1 iTBS of the hV6A did not induce changes in behavior RT or the Stroop effect

We found that iTBS over hV6A did not affect the overall level of attention as measured by the Stroop effect. The goal-directed reaching action is a hand-object interaction that occurs in a complex and changing external world, where the motor plan must be flexibly adjusted by shifting attention to a new location (attentional reorientation), in response to an unexpected change in the target’s location (Sulpizio et al., 2023). The effect of hV6A on interaction between attentional reorientation and motor performance has been discussed using the cue validity paradigm (Breveglieri et al., 2023a; Breveglieri et al., 2025; Ciavarro et al., 2013). Our study are more concerned with the effect of the left parietal iTBS, or the right parietal lobe influenced by the left parietal iTBS (the key node of the right-side advantage dorsal attention network), on the overall level of attention. The Stroop test related to attention control requires the participation of the frontoparietal network. Due to our experimental design, in which the reach task with only valid cues and the attentional test were measured separately, and the interpretation of the results should be cautious about the interaction of reach and attention.

The changes in total RT in our study are in line with the study results of Ciavarro et al. (2013) and Verhagen et al. (2013). Compared with sham rTMS, Ciavarro et al. (2013) reported that the valid trials reaction RT and reaching RT did not change after rTMS of the left hV6A. With regard to Verhagen’s experiment (Verhagen et al., 2013) on grasping actions, neither the reaction nor movement times were significantly changed. The author explained that adequate experimental design sensitivity for capturing behavioral consequences, but there was no significant outcome because the dorsolateral parietal lobe compensated for a transient SPOC (including hV6A) perturbation by TMS (Verhagen et al., 2013). In addition to the possible complementation of the other dorsal parietal lobe associated with reaching, the absence of RT results may also be related to the fact that we reduced the difficulty of the experiment in order to control the visual input. Reaching may seem like a daily behavior, it actually involves a complex visuomotor interaction, in other words, eye-hand coordination (Sulpizio et al., 2023). RT has been used to explain the overall reaching performance after hV6A stimulation (Kunimura et al., 2020), and the influence of visual input on RT cannot be ignored. Many studies on visual impairment have found that RT is strongly influenced by ‘whether the target object can be seen’ and the quality of ‘seeing’ during eye-hand coordination tasks (Pardhan et al., 2012; Grant and Conway, 2015; Chen and Huang, 2016). Different visual input settings in hV6A studies may improve the difficulty of the reaching task, see the settings of Verhagen et al. (2008) and Verhagen et al. (2013) studies, may induce larger errors. On the other hand, acting as a bimodal visual and somatosensory comparator, the visual field of hV6A perfectly matches the region of space that the contralateral arm reaching, and its mostly proprioceptive inputs from the shoulder, elbow and arm (Pitzalis et al., 2015). hV6A continuously updates the motor output and has been implicated in covert attentional shifts to adjust motor plan (Breveglieri et al., 2023a; Ciavarro et al., 2013). Therefore the effect of hV6A is not only reflected roughly in RT, but also in other higher order motoric dimensions, more detailed classifications such as arrival error, depth (Breveglieri et al., 2021a; Devi et al., 2024), specific motor phase (onset peak time) (Kunimura et al., 2020) and trajectories (Breveglieri et al., 2023a; Della-Maggiore et al., 2004; Torres et al., 2010; Beazley et al., 2024).

### 4.2 PPC-PPC functional connectivity during right-hand reaching tasks was increased after iTBS of the hV6A

Compared to sham iTBS, the alpha and beta bands functional connectivity of PPCs at rest was increased more after real hV6A iTBS, but not significantly. This fact indicates a real neural aftereffect of hV6A TBS based on strong connections in the corpus callosum, it is worth noting that the distance between left and right hV6A is only about 2 cm. As sham iTBS also induces similar but smaller changes, it may be that TMS induces indirect brain responses through auditory and somatosensory stimulation, as reported by Capotosto et al. (2012). As additional evidence, the brain region covered by such functional connectivity changes after sham iTBS is roughly around the stimulussite.

Interestingly, our current study describes that hV6A iTBS significantly affects the alpha band functional connectivity of LPPC-RPPC during right-hand reaching tasks. Task-related alpha rhythm changes after TMS have also been found in some TMS-EEG studies of PPC (Verhagen et al., 2013; Capotosto et al., 2012). Capotosto et al. (2012) reported that TMS over the PPC causally interferes behaviorally results and alpha rhythmic correlates of spatial attention. In the study of Verhagen et al. (2013), TMS also affected SPOC(including hV6A) alpha oscillations but did not disrupt behavioral performance, in addition sensorimotor complexity provided by task configuration was found to modulate SPOC alpha oscillations, and it can be demonstrated that alpha oscillations are related to the processing of motor parameters encoded by SPOC according to the task configuration. Alpha oscillations changes are driven by the hV6A function. Parietal lobe research is a promising area in stroke rehabilitation; the functional implications of altered posterior parietal activation are unclear and may be involved in maladaptive processes (Reibelt et al., 2023), but functional connectivity, including the frontal–parietal network and bilateral parietal lobes, has been found to have better functional implications (Hensel et al., 2023; Nyffeler, 2019). Bilateral PPC modulation after rTMS over the right parietal cortex and general functional outcome have been reported to improve (Nyffeler, 2019). Meanwhile, the aftereffect of rTMS largely relies on the complete corpus callosum between the PPCs, which emphasizes the value of the functional role of PPC-PPC interaction based on the corpus callosum. In addition, the alteration in neural functional specificity was similar to studies of Hensel (Hensel et al., 2023), indicating that survivors with good hand motor outcomes had stronger anterior intraparietal sulcus (aIPS) interhemispheric connectivity, a conclusion confirmed by functional magnetic resonance imaging and online rTMS. Indeed, aIPS is the key node of anterior-lateral neural circuits with functional relevance to the hand.

There is even an imbalance between the hemispheres of the parietal lobe. Koch’s experiment using trifocal transcranial magnetic stimulation found that the right PPC in healthy people could inhibit the MEP of the left PPC-M1, but the left PPC did not show such a manifestation. These changes are mediated by the corpus callosum (Koch et al., 2011). This might explain why left-hand tasks did not show the same result as the other states. Due to this imbalance between PPCs, iTBS of the left V6A caused no change in functional connectivity when subjects performed left-hand tasks. The stronger inhibition of RPPC against LPPC-LM1 counteracted the effect of iTBS, possibly due to the dominance of RPPC-RM1 with the left-hand reaching action.

The current study describes that iTBS of the hV6A did not affect the functional connectivity of PPC-M1, which presumably needs to take care of the functional context of neural activity during parietal lobe stimulation. Because of the indirect anatomy of the hV6A-M1, simple brain function regulation is far from enough. Goldenkoff demonstrated that both parietal and occipital cortex (not area hV6A) iTBS during grasping increased motor cortical excitability and improved motor performance compared to stimulation during rest (Goldenkoff et al., 2023). iTBS in the activation of motor functional networks can increase the excitability of the downstream motor cortex, improve motor performance, and “amplify” the induction of neuroplasticity between different cortices.

### 4.3 Network efficiency during right-hand reaching tasks was increased after hV6A iTBS

Small-worldness is a meaningful property of the reaching network efficiency in previous studies (Storti et al., 2016; Caliandro et al., 2021; Athanasiou et al., 2018). Above all, small-worldness responds better to changes in normal brain aging than other global properties and precedes anatomical changes (Onoda and Yamaguchi, 2013). Our experiments found that iTBS over the hV6A improved the small-worldness of right-hand reaching tasks. That is, the brain network performed the specified tasks ‘more efficiently’. It is well known that the parietal lobe, which belongs to the sensorimotor cortex, can integrate real-time information from different brain regions (e.g., temporal lobe, occipital lobe) to complete each action more accurately. This suggests that hV6A can serve as a key regulator of the reaching-related brain network and that it plays a role consistent with the properties of the region in which it is located. hV6A may even become a target for delaying the functional decline caused by brain aging (Goldenkoff et al., 2021).

### 4.4 Limitations

To the best of our knowledge, there are no experimental results of hV6A and non-invasive brain regulation in the elderly. This study did not include the elderly population. Caution should be applied to the elderly and clinical populations.

The stimulation intensity(80%AMT) in our study is similar to the landmark research by Huang et al. (2005), 80% AMT (Mioli et al., 2018; Abellaneda-Pérez et al., 2019) and 80% RMT (Gan et al., 2019; He et al., 2013) have been reported in parietal TMS studies. The choice of stimulation intensity has always been controversial and there is no gold standard, which makes sense because motor thresholds do not fully represent excitability in non-motor brain regions, although recent research suggests otherwise (Phylactou et al., 2024). According to previous studies on parietal and parieto-occipital TBS, the following conditions have been found for the choice of the stimulation intensity:(1) fixation of the stimulator output between 40 and 60% (Vesia et al., 2010; Goldenkoff et al., 2023; Whybird et al., 2021; Burke et al., 2013); (2) 80% of the adjusted AMT based on the scalp-cortex distance (Goldenkoff et al., 2023) [for details, see Stokes et al. (2005)]; (3) phosphene threshold measured through the visual cortex (Moretti et al., 2022; Stewart et al., 2001; Boroojerdi et al., 2002).

### 4.5 Clinical implications

The novelty of the current study is that we used a new neurophysiological tool (EEG) to investigate the functional connectivity (PPC-M1 and PPC-PPC) and network efficiency after iTBS over the left hV6A during task states. Previous studies focused on the parieto-frontal network by dual-site paired-pulse transcranial magnetic stimulation. Meanwhile, the modulation was based on MRI. Our results favor the functionality of hV6A in goal-directed arm movements. Thus, targeting the left hV6A may be an important median site for PPC modulation in healthy elders and those with brain injuries, thereby improving the aforementioned abilities related to the quality of life. New protocols of non-invasive precise brain regulation over the hV6A to enhance parieto-frontal network and bilateral PPC in stroke and other neurological disorders with upper limb dysfunction will be the object of future studies.

## 5 Conclusion

This is the first study to use EEG to investigate the functional connectivity and network efficiency during reaching tasks after MRI-based iTBS of the left hV6A. The result suggests that hV6A is a potential target for iTBS modulation to improve upper limb function.

## Data Availability

The original contributions presented in the study are included in the article/supplementary material, further inquiries can be directed to the corresponding authors.
